# Relationship between the endothelial dysfunction and the expression of the β1-subunit of BK channels in a non-hypertensive sleep apnea group

**DOI:** 10.1371/journal.pone.0217138

**Published:** 2019-06-19

**Authors:** Candela Caballero-Eraso, Rocío Muñoz-Hernández, María Isabel Asensio Cruz, Rafael Moreno Luna, Carmen Carmona Bernal, Jose Luis López-Campos, Pablo Stiefel, Ángeles Sánchez Armengol

**Affiliations:** 1 Unidad Médico-Quirúrgica de Enfermedades Respiratorias, Instituto de Biomedicina de Sevilla (IBiS), Hospital Universitario Virgen del Rocío/Universidad de Sevilla, Seville, Spain; 2 Centro de Investigación Biomédica en Red de Enfermedades Respiratorias (CIBERES), Instituto de Salud Carlos III, Madrid, Spain; 3 Laboratorio de Hipertensión Arterial e Hipercolesterolemia, Instituto de Biomedicina de Sevilla (IBiS), Hospital Universitario Virgen del Rocío/CSIC/Universidad de Sevilla, Sevilla, Spain; 4 Laboratorio de Investigación clínica y traslacional en enfermedades hepáticas y digestivas, Instituto de Biomedicina de Sevilla (IBiS), Hospital Universitario Virgen del Rocío/CSIC/Universidad de Sevilla, Sevilla, Spain; 5 Laboratory of Neuroinflammation, Hospital Nacional de Paraplejicos, SESCAM, Toledo, Spain; 6 Unidad Clínico-Experimental de Riesgo Vascular (UCERV-UCAMI), Hospital Universitario Virgen del Rocío, Sevilla, Spain; Harper University Hospital, UNITED STATES

## Abstract

**Study objectives:**

Vascular damage must be diagnosed early in patients with hypertension. In this regard, endothelial dysfunction (ED) is an early sign of vascular disease and a predictor of cardiovascular diseases. In obstructive sleep apnea (OSA), intermittent hypoxia triggers ED, but mechanisms are not clear. In this context, it has been described that BK channels regulates arterial tone and that chronic and intermittent hypoxia downregulates the expression of the BK channel β1-subunit facilitating vasoconstriction. Thus, we investigated the relationship among hypoxemia, ED, and mRNA expression of the β1-subunit in patients with severe OSA. We aimed to assess (1) ED in non-hypertensive patients with OSA using laser-Doppler flowmetry, (2) BK β1-subunit mRNA expression, and (3) the impact of continuous positive airway pressure (CPAP) treatment on ED and β1-subunit regulation.

**Methods:**

OSA patients underwent 24-hour blood pressure monitoring to exclude hypertension. Laser-Doppler flowmetry was performed to assess ED, and β1-subunit mRNA expression was evaluated using a blood test of peripheral blood leukocytes at baseline and after 3 months of CPAP treatment.

**Results:**

In normotensive patients with OSA, endothelial function correlated with the severity of OSA. CPAP improved endothelial function in normotensive OSA patients and the speed of the arterial response was significantly correlated with β1-subunit mRNA expression. β1-subunit mRNA expression at baseline correlated inversely with its change after CPAP.

**Conclusions:**

Sleep apnea is related to ED in normotensive patients with OSA. CPAP therapy improves endothelial function and regulates β1-subunit mRNA expression.

## Introduction

The European Society of Hypertension and European Society of Cardiology (ESH/ESC) guidelines emphasize that subclinical vascular organ damage must be identified at an early, asymptomatic stage, because subclinical organ damage constitutes an intermediate stage in the continuum of vascular diseases such as hypertension. Similarly, early-stage organ damage is indicated by endothelial dysfunction (ED), which is currently considered one of the earliest signs of vascular disease and atherosclerosis, and it is a strong predictor of hypertension and other cardiovascular diseases [1–3].

Emerging evidence indicates that obstructive sleep apnea (OSA), a common condition that causes upper airway collapse and intermittent hypoxia during sleep, can lead to ED [4–7]. Several studies have reported that OSA patients present altered endothelial function and an increase in cardiovascular morbidity [8–11], and that treatment with continuous positive airway pressure (CPAP) might improve ED [6,12]. In fact, a meta-analysis published in 2017 showed that patients with OSA presented poor endothelial function, as indicated by flow-mediated dilatation, and that this may contribute to atherosclerosis development [13]. However, the mechanisms by which OSA might induce ED are not completely understood [11]. It may be that repetitive airway collapse during sleep causes intermittent hypoxia, triggering free radical production and stimulating the activation of transcription factors that cause a cascade of pathophysiological mechanisms, including vasoconstriction, increased vascular permeability, an inflammatory and prothrombotic state, atherosclerosis, platelet aggregation, and thrombosis [14–16].

The large-conductance, calcium-activated potassium channel, also called the BK or Maxi-K^+^ channel, is most abundantly expressed in the plasma membrane of vascular smooth muscle cells and regulates the polarity of these cells [17]. Activation of the BK channel causes it to open and thus leads to K^+^ release, resulting in membrane repolarization–hyperpolarization and consequent arterial smooth muscle relaxation. Additionally, BK channels regulate nitric oxide-mediated vasodilatation; they are crucial for normal endothelial function and play a significant role in hypertension development [18–22]. Interestingly, hypoxia downregulates expression of the BK β1-subunit in rat myocytes [23], and one study showed that hypoxia-mediated downregulation of this subunit in human peripheral blood leukocytes (PBLs) reduced the vasodilator effect mediated by the BK channels. For this reason, the BK β1-subunit has been proposed as a potential mechanism in the development of hypoxia-associated hypertension [24].

At present, only one study has analyzed the relationships among hypoxemia, arterial blood pressure (BP), and the expression of the BK β1-subunit in patients who have severe OSA without hypertension. The study found that mRNA expression of the β1-subunit in PBLs from patients with correlated to both BP and nocturnal minimum oxygen saturation (SpO_2_) levels, and that treatment of the intermittent hypoxia with continuous positive airway pressure (CPAP) was accompanied by an increase in β1-subunit mRNA expression and a decrease in BP [24]. These results suggested that the BK β1-subunit plays a role in vascular deregulation in OSA. However, data are lacking regarding the relationship among intermittent hypoxia, endothelial function, and expression of the β1-subunit in PBLs in normotensive patients with OSA.

In the present study, we hypothesized that, in normotensive patients with OSA, OSA severity is correlated with endothelial function, that mRNA expression of the BK β1-subunit is correlated with endothelial function, and that CPAP improves endothelial function and regulates mRNA expression of the BK β1-subunit in these patients. To examine the vascular status in normotensive patients with OSA, we performed laser-Doppler flowmetry and analyzed mRNA expression of the BK β1-subunit in circulating leukocytes at baseline and after 3 months of CPAP treatment.

## Material and methods

### Subjects

In this prospective, observational trial, we recruited consecutive patients of both genders who had non-hypertensive, CPAP-naïve OSA indicating CPAP treatment. These patients are part of a bigger population of 82 subjects (51 men and 31 women). From these 82 patients, β1-subunit was determined in 66 (42 men and 24 women). In the other 16 patients, was not possible to determine the β1-subunit because of some technics problems with the blood samples. Finally, we have included in the study the 21 OSA patients that were not hypertensive and with the β1-subunit determination. OSA was defined as the presence of AHI >15. The recruitment process followed national guidelines [25]. The exclusion criteria were as follows: (1) hypertension, (2) daytime hypoxemia (pO_2_ < 70 mmHg in arterial blood, or SpO_2_ < 90%), (3) evidence of any other acute or chronic condition that, in the investigator’s opinion, could have influenced the parameters assessed in the study.

The present study was registered in the ClinicalTrials.gov database (U.S. National Institutes of Health: NCT01791270), conformed to the Helsinki declaration and was approved by the Human Research Review Committee at the Virgen del Rocío University Hospital (No. 2/2009). All participants provided their written informed consent before participating.

### Procedures

All the included patients underwent attended respiratory polygraphy using the Sibelhome plus (Sibelmed S.A., Barcelona, Spain) in the sleep laboratory. Specifically, this involved continuous recording of oronasal flow and pressure, heart rate, thoracic and abdominal respiratory movements, and SpO_2_. The polygraphy data were scored manually by a trained technician following the guidelines of the Spanish Society of Pulmonology and Thoracic Surgery [25]. Apnea was defined as any interruption in oronasal airflow that lasted more than 10 seconds. Hypopnea was defined as a 50% reduction in oronasal airflow for more than 10 seconds, associated with an oxygen desaturation of 4% or higher. The apnea–hypopnea index (AHI) was defined as the number of apneas and hypopneas per hour of recording; the oxygen desaturation index (ODI) was defined as the number of oxygen desaturations > 4% per hour of recording. The mean oxygen saturation obtained throughout the recorded period was defined as the average SpO_2_, and the percentage time spent with a SpO2 < 90% was defined as T90%.

Once the sleep study was performed, the participants were evaluated in the Hypertension Unit in the next week and a 24-hour ABPM was performed and read by trained investigators at baseline and after 3 months with CPAP, using a validated device (SpaceLabs 90207, Redmond, WA, USA) with a cuff size adapted to the patient’s arm circumference. The recommendations of the European Society of Hypertension were followed [1]. BP readings were taken every 20 minutes throughout the 24 hours. We considered an ABPM registry to be valid if at least 80% of the readings were valid. Average systolic (SBP) and diastolic (DBP) BP levels were obtained for the 24-hour, day-time and night-time periods. BP load was defined as the percentage of readings above the normal BP limit. Mean arterial pressure (MAP) was calculated as follows: MAP = DBP+(1/3[SBP-DBP]).A BP dipper pattern (normal circadian pattern) was defined as a reduction of 10% to 20% in systolic BP during sleep time at night with respect to BP levels during the day; any other day-sleep BP pattern was defined as non-dipper.

Hypertension was diagnosed in accordance with the ESH/ESC guidelines (systolic and diastolic higher than 130/80,135/85,120/70 mmHg for the periods of 24 hours, day and night, respectively.

The ABPM was performed in a patient’s routine day. Regarding the use of diaries and the schedule of sleep hours, patients were asked to record the different situations throughout the day that might modify the blood pressure data (physical activity, stress situations, as well as the time they have went to sleep and woke up).

The indications for CPAP were defined according to the guidelines of the Spanish Society of Pulmonology and Thoracic Surgery guidelines [25]. The optimal fixed CPAP pressure was titrated at the patients’ homes by trained personnel based on a visual evaluation of the raw data from an overnight recording using an auto CPAP device (REMstar Auto; Philips Respironics, Pennsylvania, USA). OSA patients were followed periodically in Sleep Medicine Unit. The time using the CPAP during the sleep periods was recorded to check adherence to the therapy throughout the study. The first evaluation after diagnosis was performed 1 week after the CPAP was established. The second visit was after 1 month of CPAP treatment. In each visit, CPAP adherence and treatment compliance as well as the side effects presented were checked in order to improve the treatment. To calculate the compliance we checked the software of the CPAP in every visit. Moreover, we have a nurse specialized in improving the CPAP adherence in OSA patients, so when more than the visit described were needed, the patient was checked in the specialized nurse office.

One of the most recently validated and innovative techniques to assess the magnitude of response is laser-Doppler flowmetry, which measures microcirculation. The technique analyzes the changes in brachial artery flow after release of a sphygmomanometer inflated to above systolic pressure. These changes are associated with endothelial function and microcirculation status. As previously described [26], we used the laser-Doppler linear Periflux System 5000 (Perimed S.A., Järfälla, Sweden) to measure the hyperemic response after ischemia in the brachial artery. The software of this system assesses many parameters that indicate the speed, intensity, and/or duration of the response. The time to latency, time to recovery, time to maximum flow, and slope reflect whether the response is fast enough. Faster responses indicate better endothelial function. The area under the curve (AUC) shows the intensity and duration of the response [26], and certain cardiovascular risk factors present a slower response than healthy controls [27]. This technique has been used in several studies to measure endothelial function in both adults [8,28] and children [29,30]. A detailed explanation of this technic has been included in supporting information “S1 Appendix”.

The mRNA expression of the BK β1-subunit was evaluated in PBLs as previously described.^22^ Briefly, total RNA from fresh human PBLs was isolated in the first hour after blood collection using the QIAamp RNA Blood Mini kit (Qiagen N.V., Hilden, Germany). Reverse transcription was then carried out using the Superscript III First-Strand Synthesis System (Invitrogen, Carlsbad, CA), in accordance with the manufacturer’s instructions. The mRNA expression of the BK β1-subunit was determined by quantitative real-time polymerase chain reaction (PCR) using the ABI PRISM 7500 Sequence Detection System (Applied Biosystems, Foster City, USA). The PCR products were analyzed using Mx3005P v2.02 software for real-time PCR. The following parameters were calculated: “pre-CPAP β1 mRNA” (baseline β1-subunit mRNA expression), “post-CPAP β1 mRNA”(β1-subunit mRNA expression after 3 months of CPAP treatment), “pre-CPAP β1- post-CPAP β1 mRNA” (difference in β1-subunit mRNA expression between baseline and after 3 months of CPAP), “post-CPAP β1 mRNA / pre-CPAP β1 mRNA” (ratio of β1-subunit mRNA expression after 3 months of CPAP to that at baseline).

### Statistical analysis

All analyses were performed using IBM SPSS Statistics for Windows software (IBM Corporation, Somers, NY, USA). Data are expressed as mean ± standard deviation. We used Spearman’s bivariate correlation test to analyze the association between quantitative variables. All p-values < 0.05 were considered statistically significant.

## Results

We enrolled 21 patients (14 men, 7 women) with non-hypertensive, CPAP-naïve OSA. Table 1 shows the age, anthropometric parameters, and respiratory polygraphy results of the patients.

**Table 1:** Anthropometric characteristics and respiratory parameters during sleep.

	*Mean ± SD*	*Minimum–Maximum*
***Age***	50.1 ± 12.2	32.0–77.0
***BMI (kg/m^2^)***	35.2 ± 4.9	25.7–44.9
***WC (cm)***	114.11 ± 10.4	92.0–130.0
***WHR***	0.9 ± 0.08	0.7–1.1
***AHI***	61.4 ± 22.2	27.9–108.8
***ODI***	59.5 ± 20.8	27.5–103.7
***T90%***	34.9 ± 28.1	1.0–91.7
***SpO_2_ mean (%)***	88.00± 6.8	69.1–94.6

BMI: body mass index. WC: waist circumference. WHR: waist-to-hip ratio. AHI: apnea hypopnea index. ODI: oxygen desaturation index. T90%: time spent below SpO2 90%. SD: standard deviation.

**Fig 1:**
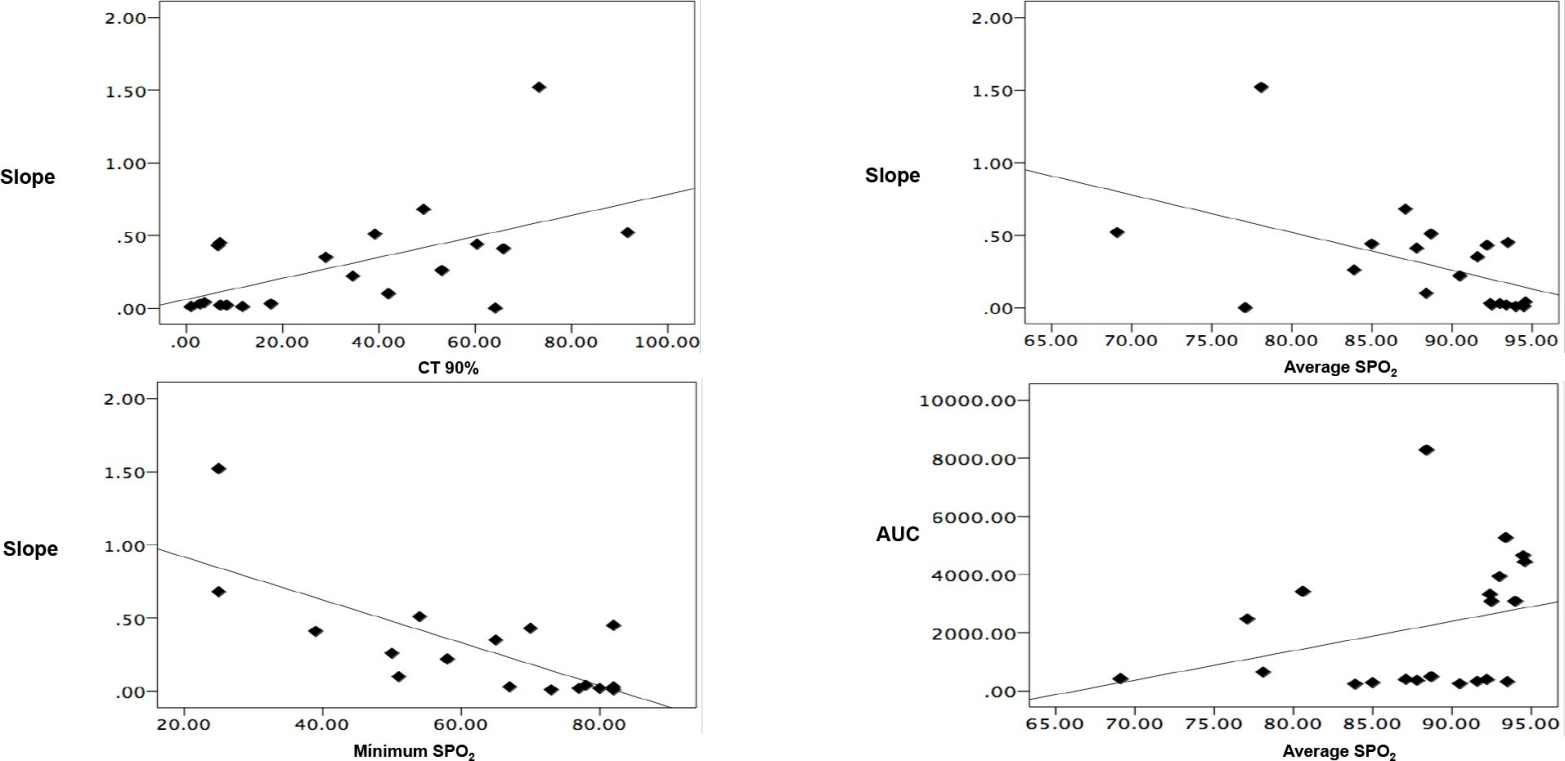
There is a relationship between the oximetric patterns of non-hypertensive OSA patients and their endothelial function. AUC: Area under the curve (perfusion units). *p < 0.05.

The patients’ oxygen saturation (SpO_2_) during sleep correlated with the responsiveness of the brachial artery to ischemia, as measured by laser-Doppler flowmetry; that is, their T90% related to the slope. Average, basal, and minimum SpO_2_ was inversely correlated with the slope, and the AUC tended towards correlation with SpO_2_ during sleep (Fig 1 and S2 Table).

Table 2 shows that CPAP treatment for 3 months improved endothelial function, as measured by laser-Doppler flowmetry.

**Table 2:** Endothelial function improves after 3 months of CPAP treatment.

	Pre-CPAP Mean ± SD	Post-CPAP Mean ± SD	p
***Hyperemia area (PU)***	1504 ± 979	1516 ± 680	r = 0,6
***AUC (PU/s)***	2.296 ± 2319	2.640 ± 2167	p = 0,01
***Slope (PU/s)***	0.32 ± 0.4	0.21 ± 0.23	p = 0,02
***Time to latency (s)***	0,99 ± 0,97	0,64 ± 0,74	p = 0,9
***Time to recovery (s)***	0,79 ± 0,60	0,94 ± 0,88	p = 0,37

AUC: area under the curve. PU: perfusion unit. s: seconds.

**Fig 2:**
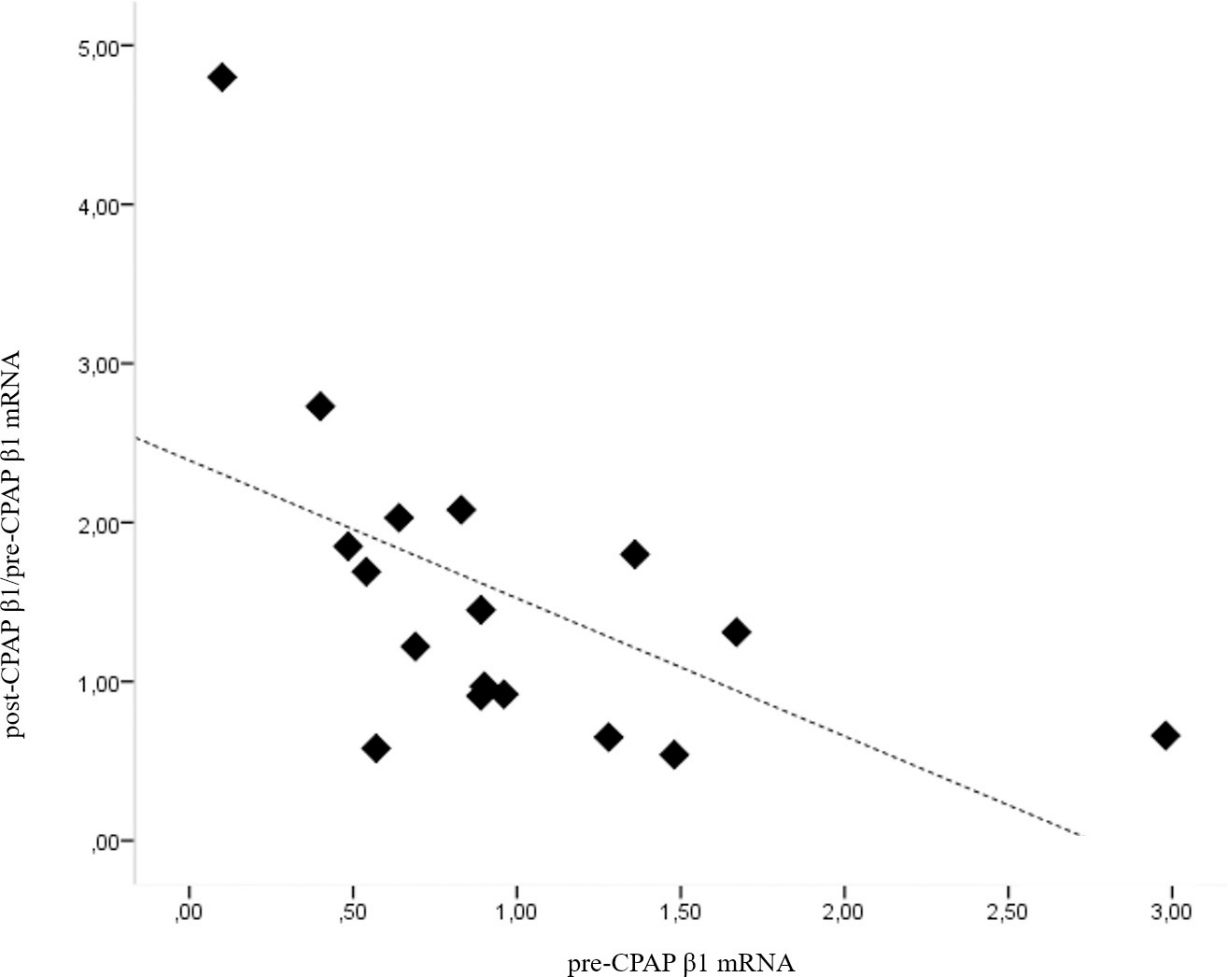
There is a correlation between β1-subunit mRNA expression at baseline and its change after 3 months of CPAP treatment. Pre-CPAP β1 mRNA: β1-subunit mRNA expression at baseline, post-CPAP β1 mRNA / pre-CPAP β1 mRNA: ratio of β1-subunit mRNA expression after 3 months of CPAP treatment to that at baseline. p = 0.009.

**Fig 3:**
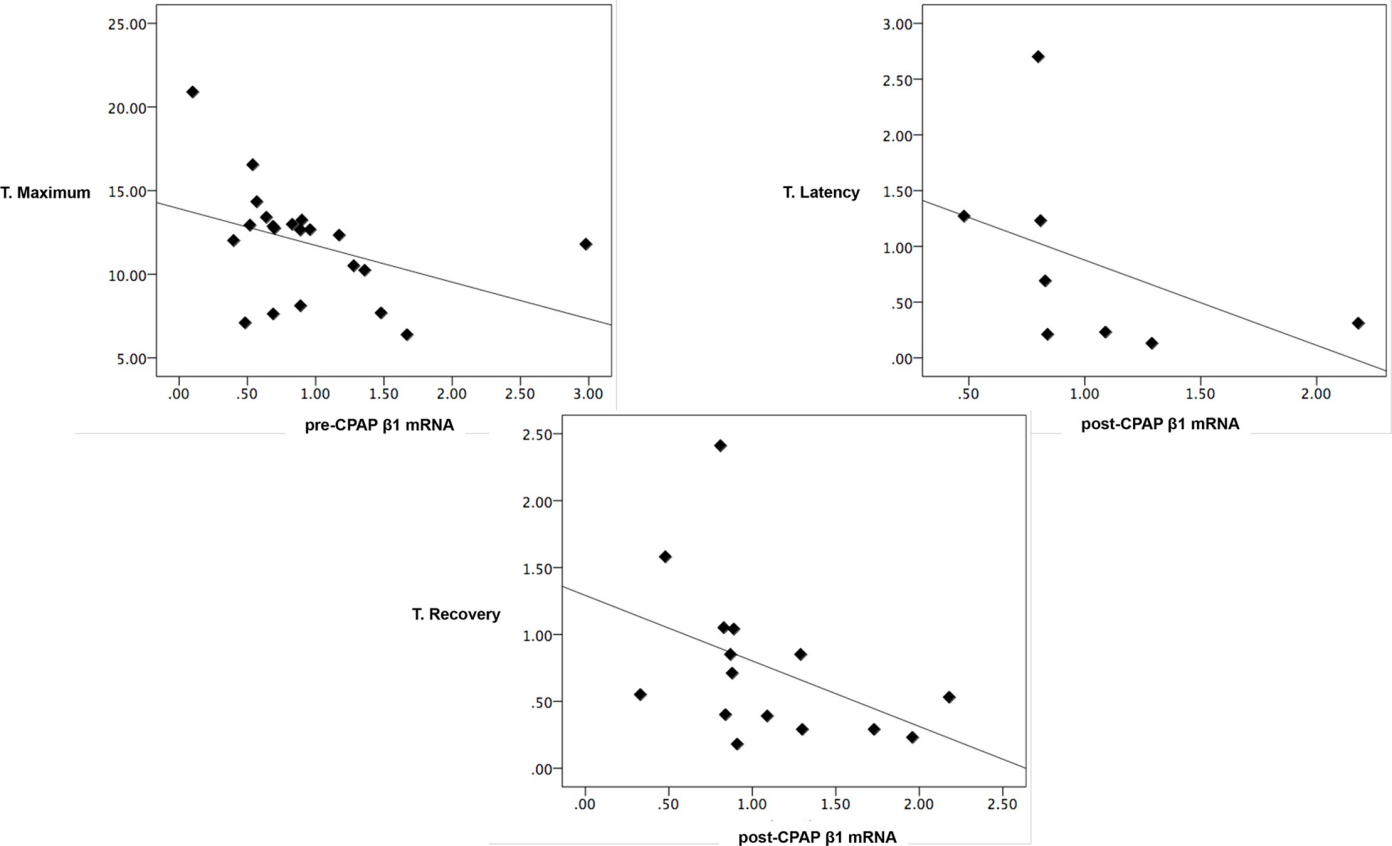
The endothelial function in non-hypertensive OSA patients was significantly correlated with β1-subunit mRNA expression. T. recovery: time to recovery. T. Latency: time to latency time. T. Maximum: time to maximum flow. pre-CPAP β1 mRNA: β1-subunit mRNA expression at baseline. post-CPAP β1 mRNA: β1-subunit mRNA expression after 3 months of CPAP treatment. * p<0.05.

No significant relationship was found between β1-subunit mRNA expression at baseline and any anthropometric parameter, or with the parameters of the sleep study. However, we revealed a significant negative correlation between β1-subunit mRNA expression at baseline and its change after 3 months of CPAP treatment. That is, higher expression at baseline was correlated with a smaller increase after CPAP (R = -612, p = 0.009, Fig 2). Furthermore, the speed of the arterial response, as measured by laser-Doppler flowmetry, was significantly correlated with β1-subunit mRNA expression (Fig 3 and S3 Table). In this figure we can observe that the higher the pre-CPAP β1 mRNA expression was, the faster the artery respond to the ischemia. The same relationship between the speed of the artery response to the ischemia was observed with the post-CPAP β1 mRNA expression: the higher the post-CPAP β1 mRNA expression was, the faster was the hyperemic artery response to the ischemia.

## Discussion

Cumulative evidence supports the clear relationship of OSA with cardiovascular disease (CVD). ED is an early clinical marker of atherosclerosis, and it has become a risk marker for development of cardiovascular disease and cardiovascular disease-related mortality [1,6,27]. Furthermore, it is an important cause of cardiovascular complications in OSA. Given the impact of untreated OSA on cardiovascular events [31–35], it is crucial to emphasize the role of CPAP in the risk of CVD, with a regulation of the ED. The present study aimed to analyze the role of CPAP in the ED and in the expression of the BK β1-subunit in a group of patients with OSA.

First of all, we found a correlation between OSA severity and ED, which is an early asymptomatic stage of vascular pathology. Moreover, after 3 months of CPAP treatment, the laser-Doppler flowmetry parameters were clearly improved. In addition, the study implied a “ceiling effect” in the changes of β1-subunit mRNA expression after 3 months of CPAP treatment. That is, patients with better endothelial function at baseline presented a larger increase in β1-subunit mRNA expression after 3 months of CPAP.

In this study we included both, men and women, because the cardiovascular consequences of OSA and the effects of the CPAP differ between the genders [5]. In contrast, most studies in this field, specifically those performed in normotensive patients with OSA [6,7], have been performed in men, including the only study to describe the correlation between hypoxia, hypertension, and β1-subunit mRNA expression [24]. Moreover, in that study, many patients presented hypertension at baseline, whereas we excluded patients with hypertension in the present study to evaluate early-stage vascular damage and the impact of CPAP on reversing this damage in patients with OSA.

Recently, intermittent hypoxia has emerged as a key factor in the development of cardiovascular damage in patients with sleep apnea [36]. In the current study, we showed a correlation between oxygen saturation during sleep and ED. Specifically, SpO_2_ during sleep was inversely correlated with the slope, indicating that lower SpO_2_ during sleep was associated the slower arterial response after ischemia. Furthermore, after correcting the intermittent hypoxia in normotensive patients with OSA using the CPAP, we saw an improvement in ED. This results suggest that, in this group of OSA patients without HTN, CPAP treatment could be beneficial in order to reduce their cardiovascular risk by controlling the ED, confirming results previously described by our own and other groups [8,37].

BK channel activity is a regulator of vascular muscle cell membrane potential and an important determinant of vascular tone, and BK β1-subunit deficiency has been correlated with hypertension [18]. Specifically, both hypertension and hypoxia downregulate mRNA expression of the β1-subunit [18,23,24], suggesting that such expression could be used as a marker for hypertension [24], and that these channels constitute a potential pharmacological target to improve vasodilator function in cardiovascular pathologies [38]. On this note, Navarro-Antolín et al. reported that minimum oxyhemoglobin saturation during sleep, a sporadic measurement that does not reflect the oximetric pattern of the patient during sleep, was related to β1-subunit mRNA expression. However, we found no relationship between the subunit and respiratory parameters during sleep, perhaps because we used a different population: Navarro-Antolín et al. only recruited men, some of whom were hypertensive at baseline, while we included both men and women and none were hypertensive.

We found a significant negative correlation between the mRNA expression of the β1-subunit at baseline and its changes after CPAP treatment. This result suggests that β1-subunit expression presents a “ceiling effect,” such that higher baseline expression of the β1-subunit is associated with smaller increases after CPAP. This may imply that the β1-subunit adapts its own expression in accordance with vascular tone. Accordingly, better endothelial function at baseline conferred a greater capacity to increase expression of the β1-subunit after CPAP treatment. Increases in β1-subunit expression after CPAP may improve cardiovascular outcomes in patients with OSA, as indicated by the improvement in ED found in the present study.

The most important limitation of this study is the small sample size. The fact that we include only non-hypertensive OSA patients and due to the high prevalence of HTN in mild-severe OSA, provoke the small sample size of this study. The lack of a control group (OSA patients without CPAP) is an additional limitation. The reasons for not including this group are the following: in our study, indications for CPAP were defined according to the guidelines of the Spanish Society of Pulmonology and Thoracic Surgery guidelines [25]. According to this guideline, patients with AHI ≥ 30 with OSA symptoms and/or cardiovascular morbidity must be treated with CPAP, given the risk of develop cardiovascular disease such as stroke or ischemic cardiomyopathy [32]. As we describe in Table 1, our patients were diagnosed as a severe OSA (AHI = 61,4 ± 22.2), so we consider that not treating these patients with CPAP during at least 3 months it's not ethics at all.

In summary, the present study confirmed that (1) the severity of OSA is correlated with ED and (2) ED can be modified by CPAP treatment reducing the cardiovascular risk. In addition, this was the first study to describe a relationship between sleep apnea, ED, and mRNA expression of the BK β1-subunit. Finally, the study revealed that mRNA expression of the BK β1-subunit shows concordance with the AUC measured by laser-Doppler flowmetry, which may indicate that the two techniques evaluate equivalent parts of the same pathophysiological process.

## Supporting information

S1 Appendix Measurement of post-ischemic reactive hyperemia by laser-Doppler flowmetry. (PDF)

S1 Table Parameters obtained by laser-Doppler flowmetry. UP: perfusion Unit. S: Seconds.
(PDF)

S2 Table Correlation between laser Doppler flowmetry and respiratory parameters during sleep. AHI: apnea hypopnea index. ODI: oxygen desaturation index. T90%: time spent below SpO2 90%. AUC: area under the curve. PU: perfusion unit. s: seconds.
(PDF)

S3 Table The endothelial function in non-hypertensive OSA patients was significantly correlated with β1-subunit mRNA expression. AUC: area under the curve. PU: perfusion unit. s: seconds. pre-CPAP β1 mRNA: β1-subunit mRNA expression at baseline. post-CPAP β1 mRNA: β1-subunit mRNA expression after 3 months of CPAP treatment.
(PDF)

S1 Fig Set up where the laser-Doppler flowmetry is performed. (JPG)

S2 Fig Example of a test performed in a healthy control. (JPG)

S3 Fig Example off a test performed in patients with a coronary heart disease. (JPG)
