# Supplementary material for: Relationship between the endothelial dysfunction and the expression of the β1-subunit of BK channels in a non-hypertensive sleep apnea group
Source: PLoS One. 2019 Jun 19;14(6):e0217138. doi: 10.1371/journal.pone.0217138 (PMC6584007; doi:10.1371/journal.pone.0217138)
Supplement: S1 Appendix — (PDF) [file pone.0217138.s001.pdf]

S1 Appendix.

The device we used to measure the endothelial dysfunction was the Periflux System 5000 Laser Doppler Flowmeter (Perimed, Jarfalla, Stockholm, Sweden). Participants were studied in our Day Hospital, in a quiet room, with only the researcher and a nurse present. Room temperature was maintained at 22ºC.

(1) In order to avoid variability, the measurements were always taken by the same researcher. Before we start, the research explained in detail to the patient the possible symptoms that the participants may experience while the study was performed. A non-inflated BP cuff was placed on the arm and the receptor probe (only one probe was used) was placed on the forearm at 15cm from the wrist (volar surface); patients lay down on the bed and remained in a resting state for about 15–20min before beginning the test "S1Fig". (2) The cuff was then inflated 40mmHg above the systolic BP and maintained in this way for minutes. During this period, the system monitor shows how the perfusion units (PU) decrease gradually to reach the biological zero. Just after 4min, the cuff was rapidly deflated so that we could observe on the monitor how the PU rise high and rapidly above the preischemic PU values.

(2) Data on perfusion in the forearm during the time of the study were recorded by the system and we noted the point of beginning of ischemia and the point of termination of the study. Then, the software analyzed the data automatically and calculated the initial value, maximum value, slope last value, peak flow, percentage of change from the first to the maximum values, time to reach the maximum hyperemia, time to reach the half value after the maximum hyperemia, and the area of hyperemia "S2 and S3 Figs".
