# Supplementary material for: Relationship between the endothelial dysfunction and the expression of the β1-subunit of BK channels in a non-hypertensive sleep apnea group
Source: PLoS One. 2019 Jun 19;14(6):e0217138. doi: 10.1371/journal.pone.0217138 (PMC6584007; doi:10.1371/journal.pone.0217138)
Supplement: S1 Table — UP: perfusion Unit. S: Seconds. (PDF) [file pone.0217138.s002.pdf]

S1 Table

PARAMETERS	MEASURE:	Better endothelial function when:
**Hyperemia area (%)**<br>**Area under the curve *(PU/s)***	Speed<br>Intensity } of the response<br>Duration	Higher
**Slope *(PU/s)***	Speed of the response	Lower
**Latency time (s)**<br>**Recovery time(s)**<br>**Time to maximum(s)**	Speed of the response	Lower
