# Supplementary material for: Relationship between the endothelial dysfunction and the expression of the β1-subunit of BK channels in a non-hypertensive sleep apnea group
Source: PLoS One. 2019 Jun 19;14(6):e0217138. doi: 10.1371/journal.pone.0217138 (PMC6584007; doi:10.1371/journal.pone.0217138)
Supplement: S3 Table — AUC: area under the curve. PU: perfusion unit. s: seconds. pre-CPAP β1 mRNA: β1-subunit mRNA expression at baseline. post-CPAP β1 mRNA: β1-subunit mRNA expression after 3 months of CPAP treatment. (PDF) [file pone.0217138.s004.pdf]

S3 Table.

	AHI	ODI	T90%	Basal SpO_2 (%)	Average SpO_2 (%)	Minimum SpO_2 (%)
**Hyperemia area (PU)**	r = 0,03<br>p = 0,91	r = 0,32<br>p = 0,88	r = 0,16<br>p = 0,46	r = - 0,27<br>p = 0,23	r = - 0,08<br>p = 0,72	r = - 0,33<br>p = 0,18
**AUC (PU/s)**	r = - 0,27<br>p = 0,20	r = -0,32<br>p = 0,15	r = -0,35<br>p = 0,11	r = 0,25<br>p = 0,27	r = 0,37<br>p = 0,09	r = 0,35<br>p = 0,16
**Slope(PU/s)**	r = 0,26<br>p = 0,26	r = 0,33<br>p = 0,15	r = 0,48<br>p = 0,03	r = - 0,42<br>p = 0,06	r = - 0,50<br>p = 0,02	r = - 0,63<br>p = 0,007
**Time to latency(s)**	r = 0,13<br>p = 0,72	r = 0,20<br>p = 0,6	r = 0,08<br>p = 0,83	r = - 0,49<br>p = 0,17	r = - 0,09<br>p = 0,93	r = - 0,07<br>p = 0,87
**Time to recovery (s)**	r = 0,31<br>p = 0,91	r = 0,02<br>p = 0,92	r = 0,13<br>p = 0,58	r = 0,05<br>p = 0,81	r = 0,005<br>p = 0,83	r = - 0,10<br>p = 0,70
**Time to Maximum (s)**	r = - 0,31<br>p = 0,89	r = 0,03<br>p = 0,89	r = 0,09<br>p = 0,67	r = -0,04<br>p = 0,83	r = -0,05<br>p = 0,8	r = -0,18<br>p = 0,48
