# Supplementary figures and images for: Relationship between the endothelial dysfunction and the expression of the β1-subunit of BK channels in a non-hypertensive sleep apnea group
Source: PLoS One. 2019 Jun 19;14(6):e0217138. doi: 10.1371/journal.pone.0217138 (PMC6584007; doi:10.1371/journal.pone.0217138)

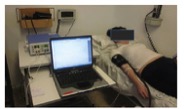

Supplement: S1 Fig — (JPG) [file pone.0217138.s005.jpg]

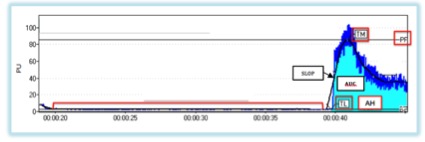

Supplement: S2 Fig — (JPG) [file pone.0217138.s006.jpg]

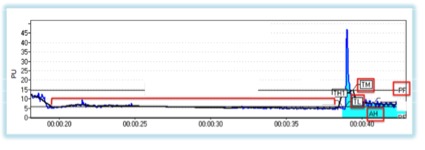

Supplement: S3 Fig — (JPG) [file pone.0217138.s007.jpg]
